# Characteristics and treatment patterns in patients with multiple myeloma in Japan: A retrospective cohort analysis

**DOI:** 10.1371/journal.pone.0315932

**Published:** 2025-01-23

**Authors:** Shinsuke Iida, Yusuke Yasutomi, Yevgeniy Samyshkin, Yi-Chen Chen, Chi-Chang Chen, Wen Shi Lee, Seok-Won Kim, Catherine McGuiness, Zifan Zhou, Simon McNamara

**Affiliations:** 1 Department of Hematology & Oncology, Nagoya City University Graduate School of Medical Sciences, Nagoya, Japan; 2 GSK Tokyo, Tokyo, Japan; 3 GSK London, London, United Kingdom; 4 GSK Singapore, Singapore, Singapore; 5 IQVIA, Wayne, Pennsylvania, United States of America; 6 IQVIA Solutions Japan G.K., Tokyo, Japan; 7 GSK, Stevenage, Hertfordshire, United Kingdom; Duke University Medical Center: Duke University Hospital, UNITED STATES OF AMERICA

## Abstract

**Background:**

Approval of proteasome inhibitors, immunomodulatory drugs, and anti-CD38 monoclonal antibodies (mAbs), such as daratumumab, has reshaped treatment patterns in patients with multiple myeloma (MM) in Japan. This retrospective study evaluated patient characteristics, treatment patterns, and trends in MM patients using Medical Data Vision, the largest electronic health records database in Japan with anonymous inpatient and outpatient health information.

**Methods:**

Patients aged ≥18 years, with ≥2 records of an MM diagnostic and disease code and ≥1 record of MM treatment between 01 April 2008 and 30 June 2023 were included. Patients starting first-line (1L) treatment on or after 01 January 2020 were categorized into the 1+L cohort; those starting second-line (2L) treatment on or after 01 January 2018 were allocated to the 2+L cohort.

**Results:**

Within the study period, 21,066 patients had an MM diagnosis, including 6,337 and 5,964 patients in the 1+L and 2+L cohorts, respectively. Median age was 74 years in both cohorts and gender distribution was similar (52.4% and 51.3% males, respectively). In the 1+L cohort, most patients (5,754/6,337; 90.8%) did not receive transplant, among whom 51.0% received 1L lenalidomide-based therapy, primarily daratumumab/lenalidomide/dexamethasone (DRd; 15.0%) or lenalidomide/dexamethasone (Rd;14.0%). In non-transplant patients, 1L DRd use increased from 6.0% in January–June 2020 to 28.0% in January–June 2023. In the 2+L cohort, 2L lenalidomide-based therapy use decreased from 65.0% in January–June 2018 to 37.0% in January–June 2023; daratumumab-based therapy increased from 14.0% to 39.0%. Retreatment with lenalidomide-, daratumumab-, and isatuximab-based therapy occurred in 44.1%, 35.2%, and 5.6% of patients, respectively.

**Conclusion:**

The high use of lenalidomide and DRd in 1L, and high rates of retreatment with lenalidomide and anti-CD38 mAbs in 2L+ indicate a substantial need for new treatment modalities that can be used in 2L+ patients who previously received lenalidomide with/without an anti-CD38 mAb therapy.

## Introduction

Multiple myeloma (MM) is a hematologic neoplasm that is characterized by proliferation of plasma cells in the bone marrow [1]. Globally, MM comprises approximately 10% of hematologic malignancies [2]. In Japan, approximately 8,200 new cases of MM and 4,500 MM-related deaths were recorded in 2020 [3]. There is limited real-world evidence of treatment patterns in patients with MM in Japan, and further investigations are needed to understand the changes in treatment patterns over time and associated burdens on these patients.

Approval of proteasome inhibitors, immunomodulatory drugs, and anti-CD38 monoclonal antibodies (mAbs), such as daratumumab and isatuximab, have reshaped MM treatment in Japan and these have been defined by the Japanese Society of Hematology (JSH) as ‘key drugs’ [4–6]. Initial treatment with these key agents, such as lenalidomide, daratumumab, or bortezomib, has improved the overall response rate (ORR) and median time to next treatment in patients with MM in Japan [7]. In addition, introduction of these agents has significantly improved survival outcomes in Japan, with 5-year overall survival (OS) rate increasing from 31.2% between 1990 and 2000 to 50.3% between 2001 and 2012 [8], especially in younger patients (5-year OS rate of 54.7% in patients aged <65 years vs. 34.9% in those aged >74 years) [1].

Despite the increasing number of available treatment options in the current therapy landscape, MM remains incurable, and most patients eventually relapse or become refractory to treatment [9]. Patients who are refractory to proteasome inhibitors and immunomodulatory drugs have particularly poor outcomes [9, 10]. Relapsed or refractory MM (RRMM) is also associated with reduced health-related quality of life driven largely by worsening pain and fatigue over time [11].

In the 2018 Japan Practical Guidelines for Hematological Malignancies from the JSH, bortezomib + lenalidomide + dexamethasone (VRd) was recommended for the treatment of transplant-eligible patients with RRMM [5]. In addition, bortezomib + cyclophosphamide + dexamethasone (VCd) and bortezomib + dexamethasone (Vd) were added as recommended regimens for VRd ineligible patients [5]. For transplant-ineligible patients, daratumumab + bortezomib + melphalan + prednisolone (D-VMp) and daratumumab + lenalidomide + dexamethasone (DRd) were recommended regimens, based on the results of the ALCYONE and MAIA studies [12–14]. These recommendations, together with the Japanese approval of daratumumab for 2L treatment in 2017 and 1L treatment in 2019 [15], have changed the treatment patterns of MM in Japan. However, few studies have investigated the changes in the use of daratumumab-based regimens in 1L and 2L treatment of MM [16].

As recommended by international guidelines, triplet and quadruplet regimens incorporating anti-CD38 mAbs, lenalidomide, and/or bortezomib are now used in earlier lines of treatment for some patients [2, 5, 17]. However, early use of these regimens limits later-line treatment options as patients become refractory to lenalidomide and daratumumab [18, 19]. Retreatment with key agents has been recommended in patients with RRMM in some situations, such as in patients who relapse more than 9–12 months after their last dose of 1L treatment [5]. However, the rationale for retreatment needs to be clarified in the real-world setting, and there is currently no consensus for optimal treatment sequencing in Japan. Therefore, there is a need to understand current patient characteristics and treatment patterns, after the local approval of daratumumab. This is particularly important when considering retreatment, where patients may be less likely to respond to repeated use of therapies with the same mechanism of action, leading to negative impacts on health outcomes and quality of life.

This retrospective study evaluated treatment patterns and trends in patients with MM using Medical Data Vision (MDV), the largest electronic health records database in Japan with anonymous health information from inpatient and outpatient sources.

## Objectives

The primary objective of this study was to describe the demographics and clinical characteristics of patients in Japan with MM who started their first line of therapy (LOT) on or after 01 January 2020 (1+L cohort), or second LOT on or after 01 January 2018 (2+L cohort), after the approval of daratumumab for 1L and 2L use in Japan in August 2019 and November 2017, respectively. The secondary objective of this study was to evaluate MM treatment patterns and sequencing in these cohorts.

## Methods

### Study design

This was a longitudinal, retrospective, observational cohort study of patients with MM in Japan identified from the MDV database. MDV collects and integrates anonymized records of discharge summaries and health insurance claims from hospitalization and outpatient visits at Japanese hospitals [20]. As of May 2024, the MDV database contains data from approximately 48 million patients from 480 hospitals, representing 28% of all Japanese hospitals that provide acute phase medical care [21]. This study focused on 1+L and 2+L MM cohorts to evaluate the treatment patterns in Japan with consideration of the approval dates for daratumumab for the 1L and 2L indications in Japan.

**Fig 1:**
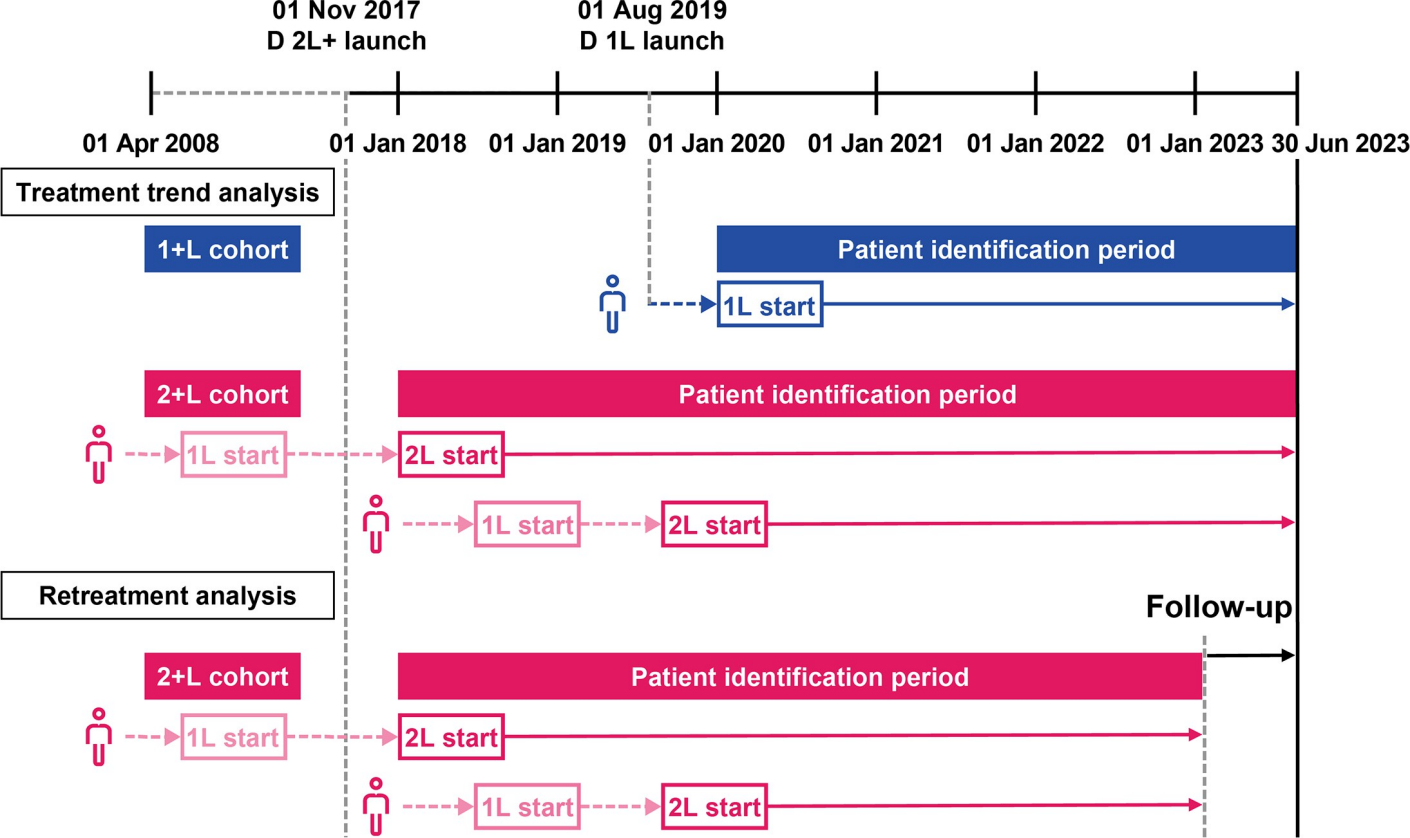
Study design, including example patient treatment journeys. 1+L cohort: patients with the start date of their 1L therapy on or after 01 January 2020; 2+L cohort: patients with the start date of their 2L therapy on or after 01 January 2018. 1L: first line; 2L: second line; D: daratumumab.

The study started on 01 April 2008 and ended on 30 June 2023. Patients who were diagnosed with MM and received one or more MM treatments were identified within the study period, and the start and end dates of their observed LOTs were determined (Fig 1). Data were accessed on 13 October 2023.

The identification periods were defined with respect to the approval dates of daratumumab for 1L (August 2019) and 2L (November 2017) treatments in Japan [15]. For the treatment trend analysis, MM treatments were reported in 6-monthly intervals. Since the aim of this analysis was to evaluate the changes in treatment selection over time, rather than exploring treatment duration or outcomes, no minimum or maximum follow-up time was considered in this analysis and patients were followed to their last observation in the MDV database. Therefore, the patient identification period was from 01 January 2020 to 30 June 2023 for the 1+L cohort, and from 01 January 2018 to 30 June 2023 for the 2+L cohort. The index date for the 1+L cohort was the start date of the first LOT (on or after 01 January 2020) to 30 June 2023, and for the 2+L cohort the start date of the second LOT (on or after 01 January 2018) to 30 June 2023 (Fig 1). For the retreatment analysis, the end date of the identification period was 31 December 2022 to allow a minimum of 6 months of follow-up prior to the end of the study period for monitoring treatment duration and outcomes. Therefore, the index date for patients in this analysis was the start date of the second LOT, on or after 01 January 2018, to 31 December 2022 (Fig 1).

The post-index period started on the index date and ended on the date of loss of data visibility, the end of the study period, or death during hospitalization, whichever occurred first. Baseline clinical characteristics and conditions of interest were evaluated during the 6-month pre-index period.

### Study population

Patients with MM in Japan who initiated 1L treatment on or after 01 January 2020 or 2L treatment on or after 01 January 2018 were included in the study. Patients aged ≥18 years with more than two records that were ≥30 days apart with a diagnostic and disease code for MM, and more than one record of MM treatment between 01 April 2008 and 30 June 2023 were eligible for inclusion in the study. Patients with more than two records that were ≥30 days apart and with a diagnosis code for lymphoma or leukemia, as well as patients with invalid age or missing gender, or more than one record for allogeneic stem cell transplant (SCT) during the study period were excluded.

### Statistical analysis

Descriptive statistics were generated for all study measures and summarized using means and standard deviations, and medians and ranges for continuous variables, and frequencies and percentages for categorical variables. Study measures were reported for the overall sample and were stratified by each LOT cohort and by subgroups of interest.

### Ethics

This study adhered to all relevant policies concerning patient privacy. There was no direct contact with patients or primary collection of individual patient data. All data were deidentified and the results of the study were provided in tabular form, ensuring that patient identification was omitted. Therefore, obtaining informed consent, ethics committee approval, or institutional review board approval was not necessary.

## Results

### Patient demographics and clinical characteristics

A total of 41,701 patients with a diagnosis and disease code for MM were identified from the MDV database during the study period. Of these, 6,337 and 5,964 patients met the criteria for inclusion in the 1+L and 2+L cohorts, respectively (Table 1).

**Table 1:** Attrition of the study sample.

Attrition of the study sample, by reason	1+L cohort^a^		2+L cohort^b^	
**Patients with ≥2 records at least 30 days apart with a diagnosis code and a disease code for MM^c^ during the study period**	41,701			
	Patients remaining, n (%)	Patients excluded, n (%)	Patients remaining, n (%)	Patients excluded, n (%)
**Patients with ≥1 record for MM treatment during the study period**	21,066 (50.5)	20,635 (49.5)	21,066 (50.5)	20,635 (49.5)
**Start date of LOT on or after the index date^d^**	7,190 (17.2)	13,876 (33.3)	6,822 (16.4)	14,244 (34.2)
**Patients ≥18 years on index date**	7,189 (17.2)	1 (<0.01)	6,820 (16.4)	2 (<0.01)
**Excluding patients with ≥2 records ≥30 days apart with a diagnosis code for lymphoma or leukemia during the study period**	6,342 (15.2)	847 (2.0)	5,968 (14.3)	852 (2.0)
**Excluding patients with ≥1 record for allogeneic stem cell transplant during the study period**	6,337 (15.2)	5 (0.01)	5,964 (14.3)	4 (0.01)
**Excluding patients with invalid age or missing gender**	6,337 (15.2)	0 (0)	5,964 (14.3)	0 (0)
**Flag patients with ≥1 record with MM diagnosis prior to index date**	6,166 (14.8)	171 (0.4)	5,937 (14.2)	27 (0.1)

^a^1+L cohort: patients with the start date of their 1L therapy on or after 01 January 2020.

^b^2+L cohort: patients with the start date of their 2L therapy on or after 01 January 2018.

^c^Diagnosis code C900 and disease codes 2030003, 8840039, 8842090, 8847250, or 8839397.

^d^Index date for 1+L: 01 January 2020, 2+L: 01 January 2018.

LOT: line of therapy; MM: multiple myeloma.

The median age of patients in both cohorts was 74 years and most patients (80.7% and 80.9% in the 1+L and 2+L cohorts, respectively) were aged ≥65 years (Table 2).The gender distribution was similar in both cohorts, with male patients making up 52.4% and 51.3% of the 1+L and 2+L cohorts, respectively. The median duration of follow-up was 431 days in the 1+L cohort and 530 days in the 2+L cohort. The median modified Deyo Charlson Comorbidity Index (CCI) was higher in the 2+L cohort compared with the 1+L cohort (3 vs. 1). Most (50.5%) patients in the 2+L cohort had a modified CCI of 3+.

**Table 2:** Patient demographics and baseline disease characteristics.

	1+L cohort^a^ N = 6,337	1+L SCT cohort n = 583	1+L non-SCT cohort n = 5,754	2+L cohort^b^ N = 5,964
**Age (years)**				
**Median**	74.0	60.0	75.0	74.0
**Age group, n (%)**				
**18–49 years**	204 (3.2)	82 (14.1)	122 (2.1)	163 (2.7)
**50–64 years**	1,018 (16.1)	343 (58.8)	675 (11.7)	974 (16.3)
**65+ years**	5,115 (80.7)	158 (27.1)	4,957 (86.2)	4,827 (80.9)
**Sex, n (%)**				
**Female**	3,017 (47.6)	250 (42.9)	2,767 (48.1)	2,906 (48.7)
**Male**	3,320 (52.4)	333 (57.1)	2,987 (51.9)	3,058 (51.3)
**Duration of follow-up, days^c^**				
**Median**	431.0	596.0	413.0	530.0
**Modified Deyo CCI (excluding malignancies)**				
**Median**	1.0	1.0	1.0	3.0
**Modified CCI categories, n (%)**				
**0**	2,143 (33.8)	271 (46.5)	1,872 (32.5)	1,004 (16.8)
**1**	1,241 (19.6)	117 (20.1)	1,124 (19.5)	1,027 (17.2)
**2**	845 (13.3)	81 (13.9)	764 (13.3)	921 (15.4)
**3+**	2,108 (33.3)	114 (19.6)	1,994 (34.7)	3,012 (50.5)

^a^1+L cohort: patients with the start date of their 1L therapy on or after 01 January 2020.

^b^2+L cohort: patients with the start date of their 2L therapy on or after 01 January 2018.

^c^Days from the index date to the end of the post-index period. The post-index period started on the index date and ended on the date of loss of data visibility, the end of the study period, or death during hospitalization, whichever occurred first.

CCI: Charlson Comorbidity Index; SCT: stem cell transplant.

In the 1+L cohort, 583 (9.2%) patients received SCT and 5,754 (90.8%) did not (non-SCT cohort). Median age was 60.0 years and 75.0 years in the SCT and non-SCT cohorts, respectively; 27.1% and 86.22% were aged ≥65 years, and 57.1% and 51.9% were male. The median modified CCI was 1 in patients who did or did not receive SCT, and the distributions of the modified CCI categories were similar between the two groups.

### Treatments at index LOT

Lenalidomide was the most commonly used treatment in the 1+L non-SCT cohort and in the overall 2+L cohort (51.4% and 53.0%, respectively, Table 3). Bortezomib-based therapy followed lenalidomide-based therapy in the 1+L non-SCT cohort; use of bortezomib-based therapy was higher in the 1+L non-SCT cohort compared with the 2+L cohort (49.9% vs. 23.6%). Daratumumab was used in 33.1% of patients in the 1+L non-SCT cohort, and in 24.0% of patients in the 2+L cohort.

**Table 3:** Most commonly used MM treatments and regimens over the study period in the 1+L non-SCT and 2+L cohorts.

	1+L non-SCT cohort^a^ n = 5,754	Overall 2+L cohort^b^ N = 5,964
**MM treatments in index LOT, n (%)**		
**Lenalidomide**	2,957 (51.4)	3,159 (53.0)
**Bortezomib**	2,872 (49.9)	1,405 (23.6)
**Daratumumab**	1,904 (33.1)	1,434 (24.0)
**Melphalan**	554 (9.6)	269 (4.5)
**Cyclophosphamide**	304 (5.3)	248 (4.2)
**Pomalidomide**	211 (3.7)	875 (14.7)
**Carfilzomib**	106 (1.8)	617 (10.4)
**Ixazomib**	84 (1.5)	831 (13.9)
**Cisplatin**	61 (1.1)	17 (0.3)
**Elotuzumab**	58 (1.0)	286 (4.8)
**MM treatment regimens in index LOT, n (%)**		
**Vd**	966 (16.8)	220 (3.7)
**Rd**	786 (13.7)	872 (14.6)
**DRd**	877 (15.2)	575 (9.6)
**VRd**	586 (10.2)	356 (6.0)
**DVd**	266 (4.6)	302 (5.1)
**D-VMpd**	230 (4.0)	43 (0.7)
**Vpd**	113 (2.0)	15 (0.3)
**DRpd**	106 (1.8)	72 (1.2)
**VCd**	105 (1.8)	74 (1.2)

^a^1+L cohort: patients with the start date of their 1L therapy on or after 01 January 2020.

^b^2+L cohort: patients with the start date of their 2L therapy on or after 01 January 2018.

D-VMpd: daratumumab, bortezomib, melphalan, prednisolone, dexamethasone; DRd: daratumumab, lenalidomide, dexamethasone; DRpd: daratumumab, lenalidomide, prednisolone, dexamethasone; DVd: daratumumab, bortezomib, dexamethasone; VMpd: bortezomib, melphalan, prednisolone, dexamethasone; LOT: line of therapy; MM: multiple myeloma; Rd: lenalidomide, dexamethasone; SCT: stem cell transplant; VCd: bortezomib, cyclophosphamide, dexamethasone; Vd: bortezomib, dexamethasone; Vpd: bortezomib, prednisolone, dexamethasone; VRd: bortezomib, lenalidomide, dexamethasone.

In the 1+L SCT cohort, the most common induction therapies were bortezomib (81.0%), lenalidomide (74.1%), and daratumumab (30.4%); the most common maintenance therapies were lenalidomide (19.4%), ixazomib (11.8%), bortezomib (7.7%), and daratumumab (5.2%) (S1 Table).

Vd was the most commonly used treatment regimen in the 1+L non-SCT cohort (16.8%), followed by DRd (15.2%) and lenalidomide + dexamethasone (Rd; 13.7%) (Table 3). In the 1+L SCT cohort, VRd + high-dose (HD)-melphalan (30.7%) and DVRd + HD-melphalan (4.8%) were the most common induction/consolidation regimens; ixazomib (26.8%), Rd (19.9%), and lenalidomide (11.3%) were the most common maintenance regimens (S1 Table). In the 2+L cohort, Rd, DRd, and ixazomib + Rd (IxaRd) were the most commonly used regimens (14.6%, 9.6%, and 8.0%, respectively).

### Treatment trends over the study period

**Fig 2:**
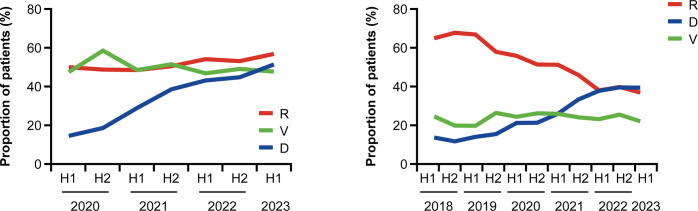
The three most commonly used MM treatments over time in the (A) 1+L non-SCT and (B) 2+L cohorts. D: daratumumab; H1: first half (01 January–30 June); H2: second half (01 July–31 December); MM: multiple myeloma; R: lenalidomide SCT: stem cell transplant; V: bortezomib.

In the 1+L non-SCT cohort, the use of daratumumab increased from 14.4% within the first interval of the January–June 2020 study period to 51.3% within the last interval of the January–June 2023 study period (Fig 2A). No major changes were observed in the use of lenalidomide- and bortezomib-based therapies over the study duration in the non-SCT 1+L cohort, nor were there major changes in the use of other less frequently used therapies (S1 Fig). In the 1+L SCT cohort, the use of bortezomib as induction therapy decreased from 84.6% in the first interval of the study period to 73.7% in the last interval of the study period, while lenalidomide use also decreased from 70.0% to 63.2% in the same periods (S2 Fig).

In the 2+L cohort, the use of lenalidomide decreased from 64.9% within January–June 2018 to 36.8% within January–June 2023 (Fig 2B). The use of daratumumab-based therapy increased from 13.5% within January–June 2018 to 39.3% within January–June 2023. No major changes were observed in the use of other treatment agents in the 2+L cohort over the study duration.

**Fig 3:**
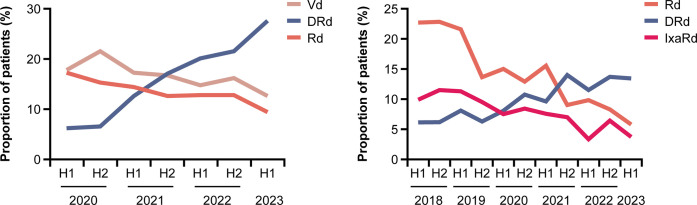
The three most commonly used treatment regimens over time in the (A) 1+L non-SCT and (B) 2+L cohorts. DRd: daratumumab, lenalidomide, dexamethasone; H1: first half (01 January–30 June); H2: second half (01 July–31 December); Rd: lenalidomide, dexamethasone; SCT: stem cell transplant; Vd: bortezomib, dexamethasone.

In the 1+L non-SCT cohort, the use of DRd increased from 6.1% within January–June 2020 to 27.6% within January–June 2023 and it was the most commonly used therapy within this period of the study (Fig 3A). There was a slight decrease in the use of Vd and Rd over the study duration. No major changes were observed in the use of treatment regimens in the 1+L SCT cohort (S3 Fig). In the 2+L cohort, the use of Rd decreased from 22.7% within January–June 2018 to 5.8% within January–June 2023 (Fig 3B). There were no clear changes in the use of other treatment regimens over the study period (S3 Fig).

### Retreatment status

Data on retreatment status were available for 5,480 patients in the 2+L cohort identified between 01 January 2018 and 31 December 2022. Demographics and baseline disease characteristics of these patients are summarized in S2 Table.

**Fig 4:**
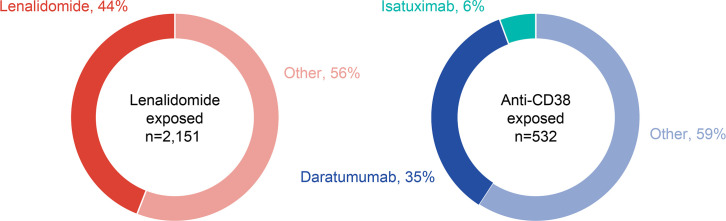
Retreatment status in the 2+L cohort.

Retreatment with lenalidomide- and daratumumab-based therapies was common in patients with exposure to ≥2 cycles of lenalidomide (n = 2,151) or anti-CD38 mAbs (n = 532) in a previous LOT (Fig 4). Retreatment with lenalidomide-based therapy was observed in 44.1% of patients with prior exposure to ≥2 cycles of lenalidomide. Of patients with prior exposure to anti-CD38 mAb therapy, 35.2% received daratumumab-based therapy and 5.6% received isatuximab-based therapy in a later LOT.

## Discussion

This real-world study used the MDV database, the largest electronic health records database in Japan, to investigate the clinical characteristics, treatment pattens and trends, and treatment sequencing in patients with MM in Japan.

In the 1+L non-SCT cohort, approximately half (51.4%) of the patients received lenalidomide-based 1L therapy and 33.1% of patients received daratumumab-based 1L therapy. This is consistent with results from a real-world study exploring treatment patterns in Japan utilizing the MDV database from 2003 to 2020, which demonstrated that Rd was the most commonly used regimen in non-transplant patients [16]. Although daratumumab is not approved as an induction therapy in Japan, 30.4% of patients in the 1+L SCT cohort were treated with daratumumab. This may be a result of patients receiving daratumumab-based therapy without the intent to undergo SCT, who were subsequently eligible for SCT after demonstrating a sufficient response to daratumumab.

Vd was the most frequently observed 1L regimen in the 1+L cohort (15.2%), followed by DRd (13.8%) and Rd (12.4%). A real-world study in Japan demonstrated an increase in 1L use of VRd between 2016 and 2020 [22], while our study found a slight downward trend from 2020 to 2023. In the 1+L non-SCT cohort, the use of DRd in 1L increased over time and was the most commonly used regimen from January–June 2023 (27.6%). This is likely due to the findings of the MAIA study, in which 1L treatment with DRd demonstrated prolonged OS (median OS not reached in either DRd or control groups; hazard ratio 0.68, 95% CI 0.53–0.86, p = 0.0013) and progression-free survival (PFS; median PFS not reached versus 34.4 months in the DRd and control groups, respectively; hazard ratio 0.53, 95% CI 0.43–0.66, p<0.0001) in transplant-ineligible patients with MM, leading to an increase in the use of DRd in the 1L setting [13].

In the 2+L cohort, the most used 2L regimens were Rd (14.6%) and DRd (9.6%). Although usage decreased over time, lenalidomide-based therapy was still commonly used in 2L (36.8%) in the last 6 months of the study (January–June 2023), closely following daratumumab-based therapy (39.3%). In addition, 2L use of pomalidomide-based therapy, an agent from the same class of drugs as lenalidomide, increased from first to last interval of the study period (10.1% to 20.3%). This change in the treatment pattern in the 2+L cohort could be attributed to the transition of lenalidomide-based therapy from 2L to 1L. Furthermore, the recent findings of the PERSEUS trial demonstrated PFS benefits when adding daratumumab to VRd induction therapy and to lenalidomide maintenance therapy in transplant-eligible patients [23], which may further increase the use of daratumumab in 1L. Together with the increasing use of DRd as 1L therapy, more patients are receiving lenalidomide and daratumumab treatment in the 1L. This results in more patients potentially becoming refractory to lenalidomide and daratumumab, limiting the number of effective available therapies at 2L and beyond and consequently leading to suboptimal outcomes [10, 24].

Of patients exposed to ≥2 cycles of lenalidomide-therapy, 44.1% were retreated with lenalidomide in a subsequent line. Two previous real-world studies in the US showed retreatment rates of 53.4% and 71.4% with lenalidomide therapy [25, 26]. These differences in retreatment rates between the findings from our study and the real-world studies in the US may be due to differences in treatment availability, standard of care, and reimbursement between Japan and the US. Of note, pomalidomide is more easily accessed after lenalidomide exposure in Japan, and DRd is recommended to be continued until disease progression or unacceptable toxicity by the JSH [5]. In a prospective study of 41 patients retreated with a lenalidomide-containing regimen, ORR (partial response or better) was achieved by significantly more patients in the 1L of lenalidomide treatment than in the 2L (68.6% vs. 14.2%, p<0.001) [27]. In addition, median PFS was significantly longer in 1L versus 2L lenalidomide treatment (15.2 vs. 4.8 months, p<0.001) [27].

Of patients with prior exposure to anti-CD38 mAb therapy, 35.2% were retreated with daratumumab and 5.6% were retreated with isatuximab, which is in line with other published real-world studies [24]. In a retrospective study in the US, retreatment with anti-CD38 mAbs was reported in 39.3% of patients [25]. In another retrospective study in the US, the ORR among patients retreated with daratumumab was 66.7% [28]; however, real-world studies in the US have reported poor prognosis associated with daratumumab retreatment [29, 30]. A real-world study in Japanese patients treated with isatuximab after daratumumab reported poor ORR (46.2%) and median PFS (5.6 months), although outcomes were significantly better in patients who were not refractory to daratumumab compared with those who were refractory (ORR 91% vs. 40%, p<0.001; median PFS 5.1 months, 95% CI 3.7–8.0 vs. not reached, 95% CI 4.1–not reached, p = 0.007) [31].

With the high use of lenalidomide and increasing use of DRd as 1L therapy, more patients are likely to become double-class refractory (refractory to proteasome inhibitor and an immunomodulatory agent) or triple-class refractory (double-class plus refractory to an anti-CD38 mAb). Treatment options are limited for these patients, and despite retreatment with lenalidomide and anti-CD38 mAbs showing poor clinical outcomes, retreatment is common in clinical practice. A recent analysis of a real-world US electronic health record database reported that over half of all patients were retreated with lenalidomide (60.8% and 63.3% of patients with double- and triple-class refractory disease, respectively) and over one-third were retreated with daratumumab (35.4% and 39.3%, respectively) despite refractoriness to the respective agents [25]. Adequately long treatment-free intervals could make retreatment more effective in some patients previously exposed to these agents. However, the median interval between the last dose of lenalidomide-based or anti-CD38-mAb-based therapy in the 1L setting and the initiation of 2L therapy can vary widely depending on individual patient factors, disease characteristics, and treatment strategies employed by physicians [25, 32]. Furthermore, delayed retreatment may not be feasible in patients whose disease progresses rapidly. Currently, the JSH guidelines recommend salvage therapy based on novel agents for patients who relapse within 9–12 months; however, as effective ‘key agents’ are used increasingly in earlier LOTs, available salvage therapy regimens with established safety/efficacy become limited, and the JSH recommends clinical trial participation where possible [5].

This study had some limitations. The MDV data are collected primarily for patient care and not for research purposes. As such, the utility of research leveraging the MDV database is limited by the completeness and accuracy of the underlying data. Data that are not recorded, that are miscoded, or that fail to accurately describe clinical diagnoses or treatment all have the potential to introduce bias. Also, the MDV data are sourced from hospital systems. Therefore, services, treatments, and laboratory results may not be captured if they are billed outside of the system collecting the data. Although the MDV database is geographically diverse, patients included in this analysis may not be representative of all patients with RRMM in Japan. However, according to data from the National Cancer Center Japan, 601 patients with multiple myeloma received initial treatment, including stem cell transplantation, in 71 facilities in the capital city of Tokyo [33]. Considering this average of 8–9 cases per facility, it can be inferred that many facilities contributed patient data to the MDV database. A further limitation is the study sample selection criteria that required the availability of baseline data, first diagnosis date, and treatments received. These criteria may bias the patient sample towards a population with more stable and frequent access to the hospitals and providers within the MDV database. Lastly, there is a potential for confounders not captured withing the MDV database to have impacted the findings of this study.

In conclusion, the high use of lenalidomide and DRd in 1L, decrease in use of lenalidomide in 2L, and high rates of retreatment with lenalidomide and anti-CD38 mAbs suggest a need for new treatment modalities for patients in the 2L and beyond, as patients become refractory to existing treatments.

## Supporting information

S1 File Plain language summary (PLS). (DOCX)

S1 Fig Most commonly used MM treatments over time in the A) 1+L non-SCT and B) 2+L cohorts. (DOCX)

S2 Fig Most commonly used MM treatments in the 1+L SCT cohort over time. (A) Induction therapy; (B) Maintenance therapy.
(DOCX)

S3 Fig Most commonly used MM treatment regimens over time in the A) 1+L non-SCT and B) 2+L cohorts. (DOCX)

S1 Table Most common treatments and treatment regimens in the overall and SCT 1+L cohorts. (DOCX)

S2 Table Baseline demographics and clinical characteristics of the 2+L cohort identified between 01 January 2018 and 31 December 2022. (DOCX)
