# Supplementary material for: Characteristics and treatment patterns in patients with multiple myeloma in Japan: A retrospective cohort analysis
Source: PLoS One. 2025 Jan 23;20(1):e0315932. doi: 10.1371/journal.pone.0315932 (PMC11756803; doi:10.1371/journal.pone.0315932)
Supplement: S1 File — (DOCX) [file pone.0315932.s001.docx]

**Characteristics and treatment patterns in patients with multiple myeloma in Japan: A retrospective cohort analysis**

# Supporting information

## Plain language summary (PLS):

Why was this study done?

- Multiple myeloma is a type of blood cancer that forms from certain white blood cells known as plasma cells
- Many treatments have been developed to treat multiple myeloma, but they can stop working and the cancer can return
- Patients can sometimes be treated with the same treatment when their cancer returns, but this can be less effective
- In Japan, lenalidomide and daratumumab are two drugs that have been made available for treating multiple myeloma since 2010 and 2017, respectively
- It is unclear at what stage of their illness patients are treated with these drugs, or what other drugs they are used in combination with (known as “regimens”)

What did the researchers do?

- Medical Data Vision is a database that contains anonymous health information about patients treated in Japan
- Our study used this database to look at the characteristics of patients with multiple myeloma in Japan, what treatments they received, at what stage, and how treatments changed over time
- We identified 6,337 patients with multiple myeloma who were treated for the first time (called the first-line cohort), and 5,964 patients who had received one previous treatment and needed further treatment as their first treatment did not work or their disease returned (called the second-line cohort)

What did the researchers find?

We found that in both cohorts:

- More than half of the patients were treated with a regimen that included lenalidomide
- The proportion of patients treated with a regimen that included daratumumab increased from January 2020 to June 2023

When we looked at 5,480 patients in the second-line cohort, we found that:

- 2,151 patients had been treated with lenalidomide before
  - Of these patients, almost half were treated again with lenalidomide
- 532 patients had been treated with daratumumab or a drug that kills multiple myeloma cells in the same way
  - Of these patients, around one-third were treated again with daratumumab

What do these findings mean?

- More patients are now treated with lenalidomide and/or daratumumab as their first treatment for multiple myeloma, which can make it harder to treat patients if their disease returns as these treatments may become less effective when they are used again
- There is a need to develop new drugs that can work when the disease returns, so patients who have previously received lenalidomide, with or without daratumumab, can have other treatment options
