# Supplementary material for: Characteristics and treatment patterns in patients with multiple myeloma in Japan: A retrospective cohort analysis
Source: PLoS One. 2025 Jan 23;20(1):e0315932. doi: 10.1371/journal.pone.0315932 (PMC11756803; doi:10.1371/journal.pone.0315932)
Supplement: S1 Fig — (DOCX) [file pone.0315932.s002.docx]

## Characteristics and treatment patterns in patients with multiple myeloma in Japan: A retrospective cohort analysis Supporting information

## S1 Fig. Most commonly used MM treatments over time in the A) 1+L non-SCT and B) 2+L cohorts.

**A) B)**


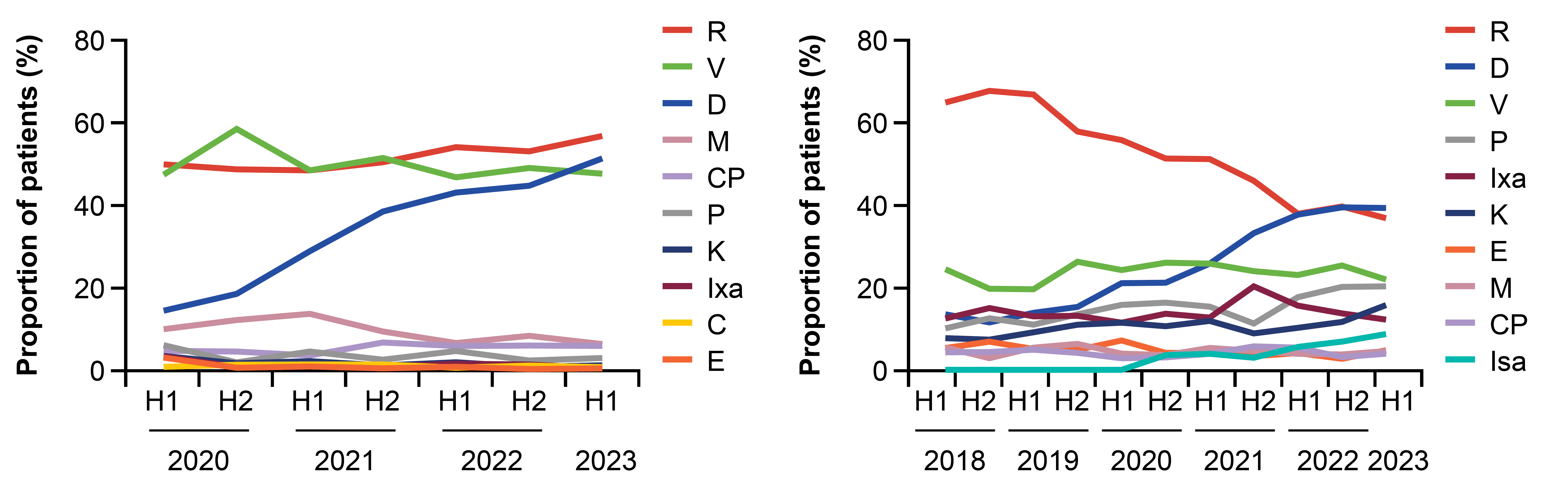


CP: cyclophosphamide; D: daratumumab; E: elotuzumab; H1: first half (01 January–30 June); H2: second half (01 July–31 December); Isa: isatuximab; Ixa: ixazomib; K: carfilzomib; M: melphalan; MM: multiple myeloma; P: pomalidomide; R: lenalidomide SCT: stem cell transplant; V: bortezomib.
