# Supplementary material for: Characteristics and treatment patterns in patients with multiple myeloma in Japan: A retrospective cohort analysis
Source: PLoS One. 2025 Jan 23;20(1):e0315932. doi: 10.1371/journal.pone.0315932 (PMC11756803; doi:10.1371/journal.pone.0315932)
Supplement: S2 Fig — (A) Induction therapy; (B) Maintenance therapy. (DOCX) [file pone.0315932.s003.docx]

**Characteristics and treatment patterns in patients with multiple myeloma in Japan: A retrospective cohort analysis**

# Supporting information

## S2 Fig. Most commonly used MM treatments in the 1+L SCT cohort over time. A) Induction therapy, B) Maintenance therapy.

**A) B)**


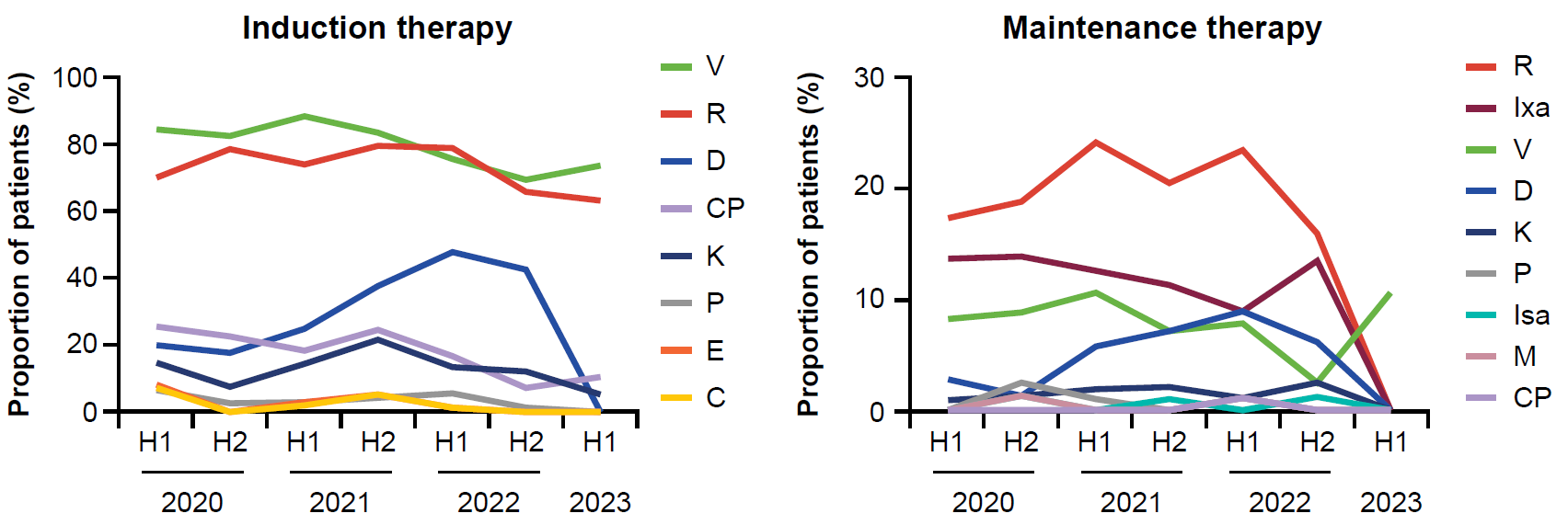


C: cisplatin; CP: cyclophosphamide; D: daratumumab; E: elotuzumab; Isa: isatuximab; Ixa: ixazomib; K: carfilzomib; M: melphalan; P: pomalidomide; R: lenalidomide; V: bortezomib
