# Supplementary material for: Characteristics and treatment patterns in patients with multiple myeloma in Japan: A retrospective cohort analysis
Source: PLoS One. 2025 Jan 23;20(1):e0315932. doi: 10.1371/journal.pone.0315932 (PMC11756803; doi:10.1371/journal.pone.0315932)
Supplement: S3 Fig — (DOCX) [file pone.0315932.s004.docx]

**Characteristics and treatment patterns in patients with multiple myeloma in Japan: A retrospective cohort analysis**

# Supporting information

**S3 Fig. Most commonly used MM treatment regimens over time in the A) 1+L non-SCT and B) 2+L cohorts.**

**A) B)**


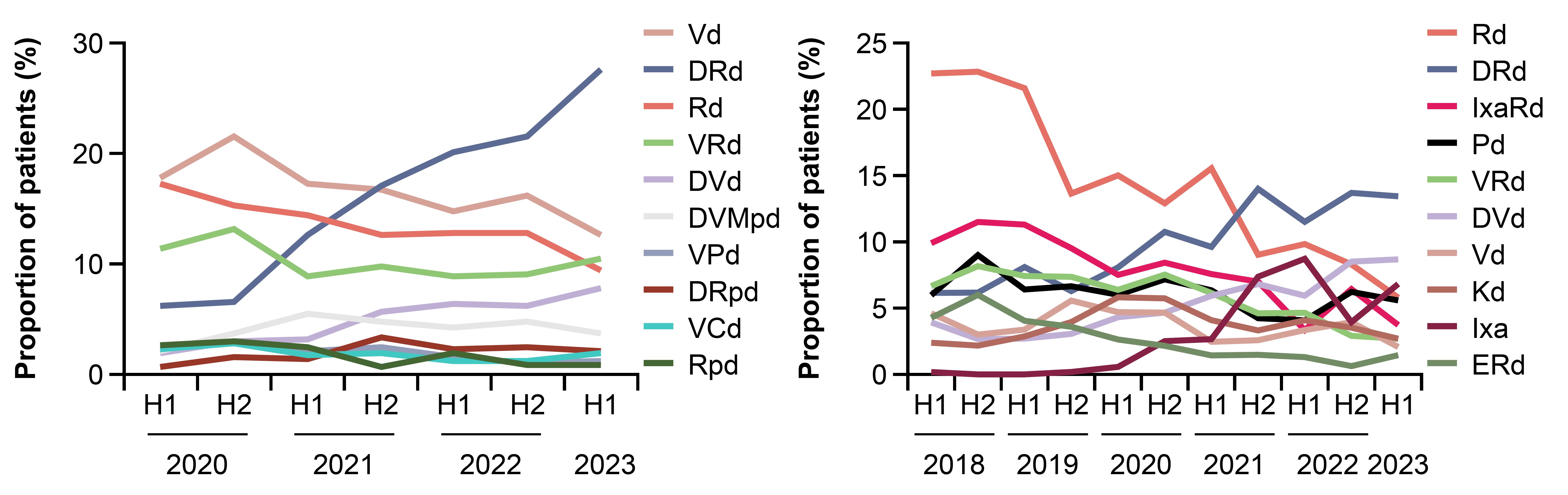


DRd: daratumumab, lenalidomide, dexamethasone; DRpd: daratumumab, lenalidomide, prednisolone, dexamethasone; DVd: daratumumab, bortezomib, dexamethasone; DVMpd: daratumumab, bortezomib, melphalan, prednisone, dexamethasone; ERd: elotuzumab, lenalidomide, dexamethasone; H1: first half (01 January–30 June); H2: second half (01 July–31 December); Ixa: ixazomib; IxaRd: ixazomib, lenalidomide, dexamethasone; Kd: carfilzomib, dexamethasone; MM: multiple myeloma; Pd: pomalidomide, low dose dexamethasone; Rd: lenalidomide, dexamethasone; Rpd: lenalidomide, prednisolone, dexamethasone; SCT: stem cell transplant; VCd: bortezomib, cyclophosphamide, dexamethasone; Vd: bortezomib, dexamethasone; VRd: bortezomib, lenalidomide, dexamethasone; Vpd: bortezomib, prednisolone, dexamethasone.
