# Supplementary material for: Characteristics and treatment patterns in patients with multiple myeloma in Japan: A retrospective cohort analysis
Source: PLoS One. 2025 Jan 23;20(1):e0315932. doi: 10.1371/journal.pone.0315932 (PMC11756803; doi:10.1371/journal.pone.0315932)
Supplement: S1 Table — (DOCX) [file pone.0315932.s005.docx]

**Characteristics and treatment patterns in patients with multiple myeloma in Japan: A retrospective cohort analysis**

# Supporting information

## S1 Table. Most common treatments and treatment regimens in the overall and SCT 1+L cohorts.

|  | Overall 1+L cohort^a^  N=6,337 | 1+L SCT cohort  n=583 | |
| --- | --- | --- | --- |
|  |  | **Induction** | **Maintenance** |
| MM treatments in index LOT, n (%) | | |  |
| Lenalidomide | 2957 (46.7) | 432 (74.1) | 113 (19.4) |
| Bortezomib | 2872 (45.3) | 472 (81.0) | 45 (7.7) |
| Daratumumab | 1904 (30.1) | 177 (30.4) | 30 (5.2) |
| Melphalan | 554 (8.7) | 582 (99.8) | 2 (0.3) |
| Cyclophosphamide | 304 (4.8) | 112 (19.2) | 1 (0.2) |
| Pomalidomide | 211 (3.3) | 22 (3.8) | 4 (0.7) |
| Carfilzomib | 106 (1.7) | 81 (13.9) | 9 (1.5) |
| Ixazomib | 84 (1.3) | 3 (0.5) | 69 (11.8) |
| Cisplatin | 61 (1.0) | 16 (2.7) | 0 |
| Elotuzumab | 58 (0.9) | 3 (0.5) | 0 |
| MM treatment regimens in index LOT, n (%) | | |  |
| Vd | 966 (15.2) | 0 | 6 (2.6) |
| DRd | 877 (13.8) | 0 | 13 (5.6) |
| Rd | 786 (12.4) | 0 | 46 (19.9) |
| VRd | 586 (9.3) | 0 | 10 (4.3) |
| DVd | 266 (4.2) | 0 | 2 (0.9) |
| D-VMpd | 230 (3.6) | 1 (0.2) | 0 |
| Vpd | 113 (1.8) | 0 | 0 |
| DRpd | 106 (1.7) | 0 | 0 |
| VCd | 105 (1.7) | 0 | 0 |
| Rpd | 99 (1.6) | 0 | 6 (2.6) |

^a^1+L cohort: patients with the start date of their 1L therapy on or after 10 January 2020
DRd: daratumumab, lenalidomide, dexamethasone; DRpd: daratumumab, lenalidomide, prednisolone, dexamethasone; DVd: daratumumab, bortezomib, dexamethasone; D-VMpd: daratumumab, bortezomib, melphalan, prednisolone, dexamethasone; LOT: line of therapy; MM: multiple myeloma; Rd: lenalidomide, dexamethasone; Rpd: lenalidomide, prednisolone, dexamethasone; VCd: bortezomib, cyclophosphamide, dexamethasone; Vd: bortezomib, dexamethasone; Vpd: bortezomib, prednisolone, dexamethasone; VRd: bortezomib, lenalidomide, dexamethasone
