# Supplementary material for: Characteristics and treatment patterns in patients with multiple myeloma in Japan: A retrospective cohort analysis
Source: PLoS One. 2025 Jan 23;20(1):e0315932. doi: 10.1371/journal.pone.0315932 (PMC11756803; doi:10.1371/journal.pone.0315932)
Supplement: S2 Table — (DOCX) [file pone.0315932.s006.docx]

**Characteristics and treatment patterns in patients with multiple myeloma in Japan: A retrospective cohort analysis**

# Supporting information

## S2 Table. Baseline demographics and clinical characteristics of the 2+L cohort identified between 01 January 2018 and 31 December 2022.

|  | 2+L cohort^a^  N=5,480 | Patients exposed to 2+ cycles of lenalidomide  n=2,151 | Patients with prior exposure to anti-CD38 mAb  n=532 |
| --- | --- | --- | --- |
| Age (years) |  |  |  |
| Median | 74.0 | 72.0 | 72.0 |
| Age group, n (%) |  |  |  |
| 18–49 years | 145 (2.7) | 92 (4.3) | 21 (4.0) |
| 50–64 years | 884 (16.1) | 420 (19.5) | 86 (16.1) |
| 65+ years | 4,451 (81.2) | 1,639 (76.2) | 425 (79.9) |
| Sex, n (%) |  |  |  |
| Female | 2,661 (48.6) | 1,049 (48.8) | 240 (45.1) |
| Male | 2,819 (51.4) | 1,102 (51.2) | 292 (54.9) |
| Duration of follow-up, days* |  |  |  |
| Median | 591.0 | 583.0 | 369.5 |
| Modified Deyo CCI (excluding malignancies) |  |  |  |
| Median | 3.0 | 2.0 | 3.0 |
| Modified CCI categories, n (%) |  |  |  |
| 0 | 911 (16.6) | 327 (15.2) | 68 (12.8) |
| 1 | 929 (17.0) | 379 (17.6) | 73 (13.7) |
| 2 | 843 (15.4) | 373 (17.3) | 92 (17.3) |
| 3+ | 2,797 (51.0) | 1,072 (49.8) | 299 (56.2) |

^a^2+L cohort: patients with the start date of their 2L therapy on or after 01 January 2018
CCI: Charlson Comorbidity Index; mAb: monoclonal antibody
